# Cardiac tamponade, an unusual and fatal complication of esophagus dilatation for benign stenosis: a case report

**DOI:** 10.1186/1757-1626-1-419

**Published:** 2008-12-24

**Authors:** Wendela L Greven, Nicole Kooij, Herman M Peters, Joost Kardux, Peter E Spronk

**Affiliations:** 1 Department of Intensive Care, Gelre Hospitals, Lukas site, Post office Box 9014, 7300 DS, Apeldoorn, The Netherlands; 2 Department of Pathology, Gelre Hospitals, Lukas site, Post office Box 9014, 7300 DS, Apeldoorn, The Netherlands; 3 Department of Radiology, Gelre Hospitals, Lukas site, Post office Box 9014, 7300 DS, Apeldoorn, The Netherlands; 4 Department of Intensive Care Medicine, Academic Medical Center, Post office box 22660, 1100 DD, Amsterdam, the Netherlands; 5 HERMES critical care group, Amsterdam, the Netherlands

## Abstract

**Introduction:**

We present a patient with a fatal late esophago-pericardial fistula three months after dilatation for benign oesophagus stenosis

**Case presentation:**

A 71-year-old caucasian male with a known benign esophagus stenosis was referred to the ICU. On arrival an asystole developed which proved to be due to a large pericardial effusion. Pericardial fluids were drained, but the patients' condition worsened and he died due to multiple organ failure. Postmortum investigation revealed an esophago-pericardial fistula.

**Conclusion:**

Causes of an acute tamponade should also be sought in semirecent events, such as manipulation to the oesophagus months before the acute critical illness.

## Introduction

Cardiac tamponade can be caused by pericardial effusion and should always be considered whenever asystole is present. Causes of these effusions are often idiopathic, but include infections (viral, bacterial, tuberculosis), malignancy, iatrogenic causes, post-myocardial infarction or trauma, uremia, collagen vascular disorders, and radiotherapy [1,2]. In the developed world the most common cause of pericardial effusion is idiopathic pericarditis (80%) [3], mostly caused by viral infections and its immunological response to it [4]. In idiopathic pericarditis, tamponade is rare (14%), but it is more common (61%) with other causes such as neoplastic effusion, tuberculosis and purulent effusion [4].

Fistula of the esophagus to the pericardial space have been described before and could cause infectious pericarditis with effusion. Among other causes of fistula formation are manipulation to the esophagus [5,6]. Esophagus dilatation is performed on a regular basis for benign stenotic conditions. We herein present a patient with a fatal late complication of this dilatation.

## Case Presentation

A 71-year-old Caucasian male, was referred to our clinic because of high fever (39.2°C). His medical history showed benign esophagus stenosis due to recurrent reflux disease and concomitant candida esophagitis. He did not take regular medication. On presentation the patient had fever and was feeling very ill, without any specific complaints. He did not smoke, drank only alcohol in weekends and had no significant family history. On physical examination the blood pressure was 80/40 mm Hg, pulse rate 115 bpm, respiratory rate 32/min, length 178 cm, weight 78 kg. Further physical examination, including cardiac chest sounds, EKG, lung sounds and abdominal examination was normal. Laboratory results showed elevated C reactive protein (252 mg/l) and a leukocytosis (16.9/nL). A chest x-ray and a regular urinary analysis did not show any infection. A sepsis of unknown origin was concluded and blood cultures were drawn. He was admitted to a nursing ward and treated with ceftriaxon (2 gr intravenous) and gentamycine (240 mg intravenous). The same night, his condition worsened and he was admitted to the intensive care unit (ICU), because of respiratory failure and hypotension. On physical examination at the ICU he still had high fever (39.1°C), a breathing frequency of 40/minute and a worsening hypotension (mean arterial pressure 51 Mm Hg). It was not possible to hear cardiac sounds and his extremities were cold. He was intubated and after placement of a central line, the initial CVP was 7 cm H2O. Intravenous fluid replacement was started as well as inotropics (dopamine 4.5 μg/kg/min; norepinephrin 0.5 up to 2.3 μg/kg/min; milrinone 0.25 μg/kg/min). The patients' condition worsened quickly and an aystole developed. Cardiopulmonary resuscitation (CPR) was started. An ultrasound of the heart showed a tamponade with pericardial effusion. Pericardial fluids were drained and his hemodynamic situation improved rapidly, although vasopressors could not be completely weaned. The patient improved at first, but within 12 hours his condition worsened again. A new ultrasound of the heart showed again pericardial effusion, in which a small catheter was placed to perform drainage. However, his clinical condition worsened further and he died a few hours later due to hemodynamic shock unresponsive to therapy.

**Figure 1:**
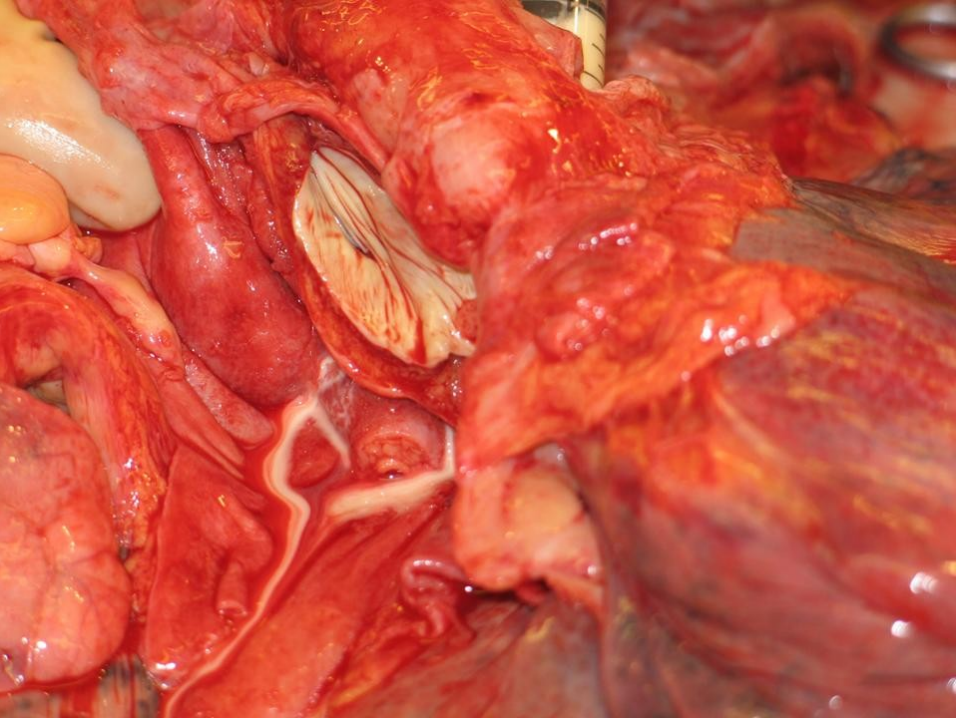
**A white liquid was used to confirm the fistular connection, i.e. within a few seconds it appeared from the esophageal origin in the pericardial space**.

Postmortum examination showed a fistula from the esophagus directly to the pericardium. A white liquid was used to confirm the fistular connection, i.e. within a few seconds it appeared from the esophageal origin in the pericardial space (figure 1). Post-mortem culture of the pericardial effusion showed candida species.

His family told that he had had complaints of unwell being and atypical thoracic pain since his last esophagoscopy with dilatation of the esophagus stenosis, three months before. Most likely he had been suffering a chronic (candida) mediastinitis since his last esophagus dilatation three months before ICU admission, which had been complicated by fistula formation to the pericardial space, resulting in a cardiac tamponade due to pericardial effusion.

## Discussion

Fistula between the esophagus or stomach and pericardium or other sites of the heart (atrial, ventricular) have been described before. Predisposing factors to fistula formation are surgery to the esophagus, such as esophagectomy with retrosternal gastric tube reconstruction [5] and Nissen fundoplication [6]. Other predisposing factors to fistula formation are esophageal diverticula [7], neoplasmata [8-10], benign ulcers [11] and foreign bodies [12]. The mechanism could consist of a fistula from the esophagus to the mediastinum first, resulting in a chronic mediastinitis, resulting in fistula formation to the pericardium of the heart or other sites in the mediastinum, such as the diaphragma [13]. Our patient had suffered of chronic candida esophagitis for some months. Moreover a dilatation was performed to the esophagus, most likely causing a trauma. So our patient not only had a porte d'entrée, but he was also colonized with a pathogen, that could cause the mediastinitis and eventually fistula formation to the pericard, causing pericarditis.

In literature described pathogens causing a pericarditis due to fistula include Candida species [5,6], and streptoccus species [7,10]. Candida pericarditis is a rare surgical emergency, usually occurring in immunocompromised hosts, antibiotic-treated patients or after pericardiotomy [14]. Esophagopericardial fistula have a very high mortality, approximately 85% [11]. In candida pericarditis, pericardiocentesis, antifungal therapy, followed by operative drainage is the therapy of choice [14]. Herein early diagnosis and prompt treatment are most important [15] as is operative closure of the fistula [11]. In our patient, due to his septic state and the rapid worsening of his condition only pericardiocentesis could be performed, which was unfortunately not sufficient to stabilize his clinical status.

## Conclusion

Esophagus-pericardial fistula have been described in varying predisposing conditions. This is the first case demonstrating cardiac tamponade due to fistula formation after esophagus dilatation for benign stenosis. This report shows that causes of an acute tamponade, should also be sought in semirecent events, such as manipulation to the oesophagus months before acute critical illness.

## Consent

Written informed consent was obtained from the patient for publication of this case report and accompanying images. A copy of the written consent is available for review by the Editor-in-Chief of this journal.

## Competing interests

The authors declare that they have no competing interests.

## Authors' contributions

WG analyzed and interpreted the patient data. WG and PS were the major contributors in writing the manuscript. All authors read and approved the final manuscript.
